# Peer Review of “COVID-19 Outcomes and Genomic Characterization of SARS-CoV-2 Isolated From Veterans in New England States: Retrospective Analysis”

**DOI:** 10.2196/35517

**Published:** 2021-12-17

**Authors:** Lei Guo

**Affiliations:** 1 Center for Reproductive Health Sciences Department of Obstetrics & Gynecology Washington University School of Medicine St.Louis, MO United States

**Keywords:** infectious disease, COVID-19, epidemiology, veteran, outcome, sequencing, genetics, virus, United States, impact, testing, severity, mortality, cohort

*This is a peer-review report submitted for the paper “COVID-19 Outcomes and Genomic Characterization of SARS-CoV-2 Isolated From Veterans in New England States: Retrospective Analysis.”*

## Round 1 Review

### General Comments

The authors presented a study [1] about the clinical and genomic characterization of COVID-19 from a veteran group. I have some questions for the authors.

1. Line 85: Authors wrote, “we recorded hospitalization status, mortality, and oxygen (O2)-requirement within 24 hours of admission.” Here, can authors clarify if they recorded each single patient’s clinical information within 24 hours of admission or they collected them from chart review? In addition, for O2, the 2 should be subscript.

2. Lines 105 and 106: The disease name should be capitalized.

3. Line 113: Authors did not provide a transition between the univariate regression and multivariate regression. Univariate analysis was simply mentioned in the first sentence without any explanation or discussion. Authors should indicate the reason why they conducted multivariate analysis (eg, univariate was not specific enough). Additionally, in general, the factors should have the first letter capitalized, for example, Age, Non-White Race.

4. Line 129: Authors wrote, “our study found that in an older cohort of veterans.” Here, older cohort could cause some confusion to some readers. When one reads the paper a few years later, he or she probably cannot understand what the older cohort is related to. Authors can add a time frame to it.

5. Line 131: Similar to point 4, authors should add the Centers for Disease Control and Prevention (CDC) report date.

6. Line 133: Authors wrote, “veterans are a unique cohort because of advanced age on average, and more comorbidities. Understanding clinical factors that impact outcomes in veterans will help clinicians risk-stratify patients with similar demographic profiles.” Many veterans could be young in some Veterans Affairs (VA) medical centers. It may be right to general veteran populations, but authors need to cite references to support this claim.

7. Line 137: Authors wrote, “in our study, age was a significant predictor for all of our outcomes and was a confounder for other variables.” Most scientific papers are written from the third point of view. Therefore, it is not common to state the study outcomes as “our outcome.” Authors should use a better phrase, such as in line 151: “This may explain the outcomes in our study.”

8. Line 138: Authors wrote, “interestingly, LTC status predicted all three of our outcomes on univariate analysis, but not on multivariate analyses. Earlier in the COVID-19 pandemic, residents of nursing homes had higher rates of infection as well as severe illness and mortality [2].” There is no transition between these two sentences. The first few sentences in the paragraph discussed age as a predictor. However, the sentence “earlier in the COVID-19 pandemic...” did not show an immediate connection with the age issue. Maybe the authors would like to express that nursing homes have older patients. If this is the case, the authors need to provide some connection or background information here.

9. Line 140: Authors wrote that “our study shows that among veterans in LTC facility, disease outcomes were not impacted by their residence status.” Here, authors should provide some discussion or reasons for their findings.

10. Line 148: Authors wrote, “our study supports data from previous reports that non-White patients are at increased risk of hospitalization but have similar peak severity and mortality outcomes [3-6].” Are these non-White patients in the United States or in other countries? This could change the dynamic and purpose of citing the reference. Please clarify.

11. Line 156: Authors concluded that, for patients with dementia, they could have a high risk of death because of biological factors. Another possibility is the lack of self-report ability in patients with dementia. As a result, they probably do not understand their body’s changes, which could delay the needed care.

12. For the Discussion section, authors may add subtitles to different issues they would like to discuss. The current writing may be a little bit confusing to some readers.

13. In the Discussion, the authors mentioned multivariate analysis of many potential risk factors as their strength. It is true that the multivariate model is a powerful tool, but it is not necessarily fit for the COVID-19 situation very well. Authors need to cite references about other cases of using the multivariate model for COVID-19 outcome analysis.

14. Figures and supplemental tables: Authors should include more details in the titles. Simply writing “genomes” or “hospitalization” in the title is not standard in scientific papers.

15. Figure 1: Authors should provide a better maximum likelihood tree. The current figure has many branches stacked to each other, barely providing any helpful information to readers.

## Round 2 Review

The authors presented an updated manuscript after taking the reviewers’ suggestions. I have a few minor comments.

1. Authors added reference [7] but did not indicate or cite it in the paper. I guess it should be listed here: “which has been frequently used in COVID-19 literature [8-11].”

2. Authors wrote, “this study included all veterans who tested positive for COVID-19 from April 8, 2020, to September 16, 2020 at one of the six New England VA hospitals.” Previously authors wrote, “Connecticut had been entrusted with testing for SARS-CoV-2 for all six VA healthcare centers.” Does this mean the patients enrolled in this study are from one of six VA hospitals, or they are from all six hospitals?

3. Authors wrote, “the CDC provides a list of chronic medical conditions (May 2021) that predispose individuals to severe illness from SARS-CoV-2 infection [12], but >75% of United States adults fall under a high-risk category [13].” In general, if the word “but” is in the sentence, readers will pay attention to the words following “but,” which means the first part may not be important or critical. Authors can kindly use another connection word.

4. In the Abstract, the authors wrote “Multiple SARS-CoV-2 lineages were distributed in patients in New England early in the COVID-19 era, mostly related to viruses from New York with D614G mutation.” Can the authors kindly clarify if it is New York State or New York City?

## Abbreviations

CDC: Centers for Disease Control and Prevention
VA: Veterans Affairs
